# Oscillatory contractile forces refine endothelial cell-cell interactions for continuous lumen formation governed by Heg1/Ccm1

**DOI:** 10.1007/s10456-024-09945-5

**Published:** 2024-09-09

**Authors:** Jianmin Yin, Ludovico Maggi, Cora Wiesner, Markus Affolter, Heinz-Georg Belting

**Affiliations:** Department of Cell Biology, Biozentrum, University of Basel, Spitalstrasse 41, Basel, 4056 Switzerland

**Keywords:** Krit1, Heg1, Cerebral cavernous malformations (CCMs), Endothelial cells, Oscillation, Actomyosin contractility, Lumen, Anastomosis

## Abstract

The formation and organization of complex blood vessel networks rely on various biophysical forces, yet the mechanisms governing endothelial cell-cell interactions under different mechanical inputs are not well understood. Using the dorsal longitudinal anastomotic vessel (DLAV) in zebrafish as a model, we studied the roles of multiple biophysical inputs and cerebral cavernous malformation (CCM)-related genes in angiogenesis. Our research identifies *heg1* and *krit1* (*ccm1*) as crucial for the formation of endothelial cell-cell interfaces during anastomosis. In mutants of these genes, cell-cell interfaces are entangled with fragmented apical domains. A Heg1 live reporter demonstrated that Heg1 is dynamically involved in the oscillatory constrictions along cell-cell junctions, whilst a Myosin live reporter indicated that *heg1* and *krit1* mutants lack actomyosin contractility along these junctions. In wild-type embryos, the oscillatory contractile forces at junctions refine endothelial cell-cell interactions by straightening junctions and eliminating excessive cell-cell interfaces. Conversely, in the absence of junctional contractility, the cell-cell interfaces become entangled and prone to collapse in both mutants, preventing the formation of a continuous luminal space. By restoring junctional contractility via optogenetic activation of RhoA, contorted junctions are straightened and disentangled. Additionally, haemodynamic forces complement actomyosin contractile forces in resolving entangled cell-cell interfaces in both wild-type and mutant embryos. Overall, our study reveals that oscillatory contractile forces governed by Heg1 and Krit1 are essential for maintaining proper endothelial cell-cell interfaces and thus for the formation of a continuous luminal space, which is essential to generate a functional vasculature.

**Supplementary Information:**

The online version contains supplementary material available at 10.1007/s10456-024-09945-5.

## Introduction

Biophysical forces, such as contractile forces from the actomyosin cytoskeleton and hemodynamic forces from blood flow, regulate morphogenesis and mechanotransduction pathways within endothelial cells (ECs), thereby shaping vascular networks [1–4]. Within ECs, the actomyosin cytoskeleton generates contractile forces that are essential for processes such as cell shape changes, migration, and cell-cell junction remodelling. However, little is known about how the actomyosin cytoskeleton is organized and regulated in distinct cellular compartments during the formation of the vascular network.

The development of a functional vascular network during embryogenesis necessitates both vasculogenesis and angiogenesis. Initially, individual EC progenitors aggregate to form cord-like structures, which subsequently undergo luminal opening to generate central cavities during vasculogenesis [5, 6]. As development progresses, angiogenesis becomes the predominant mechanism for vascular expansion. This dynamic process involves the sprouting of new vessels from pre-existing ones, followed by their elongation and eventual connection to adjacent sprouts or existing vessels via anastomosis [7–9]. In this study, we used the formation of the dorsal longitudinal anastomotic vessel (DLAV) in zebrafish as an experimental model to analyze the roles of the actomyosin cytoskeleton in establishing proper intercellular and apical connections during angiogenesis [10]. Specifically, we examined the roles of cerebral cavernous malformation (CCM)-related genes *heg1* and *krit1* in the precise regulation of the actomyosin cytoskeleton in ECs and the development of a functional vascular network.

CCMs are common vascular abnormalities with an estimated prevalence of 0.1–0.5% in the human population [11]. CCMs are characterized by enlarged small blood vessels with thin walls and irregular structures, leading to headaches, seizures and strokes, typically in midlife. These malformations primarily occur in brain regions with reduced blood flow. The triggers for CCM lesion formation in affected individuals remain unclear. Understanding the molecular mechanisms underlying CCM pathogenesis is essential for developing potential therapeutic interventions.

CCMs are caused by mutations in the *krit1(ccm1)*, *ccm2* and *pdcd10(ccm3)* genes [12]. In zebrafish embryos, loss of *krit1* and *ccm2* leads to the dilation of major vessels accompanied by the progressive spreading of endothelial cells and thinning of vessel walls [13]. ECs lacking Krit1 exhibit heightened RhoA activation and amplified activity in the downstream effectors ROCK1 and ROCK2, resulting in increased actomyosin-mediated contractility and the accumulation of stress fibers [14–17]. More specifically, another study revealed that Krit1 regulates ROCK1 and ROCK2 differently [18]. Krit1 recruits ROCK2 to cell-cell junctions but inhibits ROCK1-dependent actin stress fibers to ensure a proper balance between cell-extracellular matrix adhesions and cell-cell junctions. The transmembrane protein Heg1 directly binds to Krit1 to stabilize endothelial cell-cell junctions [19, 20]. Heg1 deficiency in mouse embryos results in a loss of cardiac and pulmonary vascular integrity and dilated lymphatic vessel malformations [21]. Additionally, Heg1 anchors another Rap1 effector protein, Rasip1, at cell-cell contacts to maintain EC junctional integrity [19, 22]. However, the precise molecular mechanisms underlying junctional stability and integrity, as well as the coordination of different components within this complex, remain largely unknown.

In this study, we have employed advanced live imaging with molecular reporters of cell-cell junctions, apical compartments, and the actomyosin cytoskeleton to study endothelial cell-cell interactions and apical compartmentalization throughout anastomosis. We combined genetic analysis and optogenetic tools with live imaging approaches to investigate the diverse roles of the Krit1-Heg1-Rasip1 complex in regulating junctional stability and integrity. We identified oscillatory contractions along junctions in wild-type embryos, resulting in the straightening of discontinuous or zigzag-shaped junctions. Conversely, in *heg1* or *krit1* mutants, junctions became contorted due to the absence of essential actomyosin contractility along cell-cell junctions. Thus, we identified a key role for Heg1 and Krit1 in inducing oscillatory actomyosin contractility along junctions, thereby establishing proper cell-cell interactive interfaces necessary for creating interconnected luminal spaces.

## Methods

### Zebrafish strains and morpholinos

Zebrafish (*Danio rerio*) were maintained according to FELASA guidelines [23]. All experimental procedures adhered to federal guidelines and received approval from the Kantonales Veterinäramt of Kanton Basel-Stadt (1027 H, 1014HE2, 1014G). New zebrafish lines developed in this study: *Tg(UAS: Heg1-GFP)*^*ubs61*^, *Tg(UAS: GFP-Radil2a)*^*ubs62*^. Previously established zebrafish lines: *Tg(cdh5: cdh5-TFP-TENS-Venus)*^*uq11bh*^ [24], *TgKI(tjp1a-tdTomato)*^*pd1224*^ [25], *Tg(fli1a: GFP-Podxl1)*^*ncv530Tg *^ [26], *Tg(kdrl: Myl9a-GFP)*^*ip5Tg*^ [27], *Tg(fli1ep: gal4ff)*^*ubs3*^ [28], *Tg(UAS: mRuby2-UCHD)*^*ubs20*^ [29], *Tg(UAS: EGFP-hsZO-1*,*cmlc: EGFP)*^*ubs5*^ [30], *Tg(fli1a: Rasip1-scarlet-I)*^*ubs59*^ [31], *rasip1*^*ubs28*^ [32], *heg1*^*m552*^ [33] and *krit1*^*t26458*^ [13]. Transient expressions in zebrafish embryos: *fli1a: GFP*, *fli1a: RhoA-BcLOV4-mCherry*. Morpholinos (GeneTools) used were as follows: silent heart *(sih*/*tnnt2*) CATGTTTGCTCTGATCTGACACGCA [34] and standard control CCTCTTACCTCAGTTACAATTTATA.

### Molecular biology and transgenesis

Plasmids were constructed using Gibson assembly, Gateway, or restriction cloning methods, unless specified otherwise. All constructs were inserted into Tol2 vectors before injection into zebrafish embryos, unless stated otherwise. Complete information regarding plasmid cloning and transgenesis procedures can be found in Supplementary Note 1.

### Live imaging and optogenetics

Zebrafish embryos aged between 30 and 32 h post-fertilization (hpf) were dechorionated and anesthetized using 0.16 mg/ml (1×) tricaine methanesulfonate (Sigma). These embryos were then mounted in microwell dishes within 0.75% low-melting-point agarose (ROTI) and covered with E3 buffer supplemented with 1× tricaine. Live imaging and optogenetic activation were conducted using a Leica SP5 confocal microscope equipped with a ×40 water immersion objective. Optogenetic activations were performed in manually selected regions of interest using a 450 nm laser continuously during live imaging. The sequential imaging of Venus and GFP was described in a previous study [31].

### Image analysis and data analysis

Images were analyzed using ImageJ and MATLAB, employing custom-made code. Z-stacks projections were created using maximum or sum intensity projections in ImageJ. The zigzag index was measured in ImageJ using the Freehand selections and Measurement functions. The kymographs were generated in ImageJ using the Reslice function. The measurement of the area of junctional rings, the quantification of relative signal levels of Cdh5 and Myl9a, and the unrolling of Myl9a along the junctional rings were performed using MATLAB with custom-made scripts, which are available at https://github.com/Jianmin-YIN/Heg1_Krit1_Oscillatory_Contraction. Image panels were generated using the Open Microscopy Environment (OMERO). 3D movies were created with Imaris Viewer. Color coded 3D diagrams were made using the online tool Spline at https://spline.design. Statistical analysis (t-test) was conducted using MATLAB. Sample sizes were not predetermined using statistical methods.

### Immunofluorescence

For immunofluorescence of Rasip1 in zebrafish embryos, dechorionated fish embryos were fixed for 15 min at room temperature in 2% TCA in PBST. Following fixation, embryos were washed four washes with PBST and permeabilized with 0.5% Triton-X-100 in PBS at room temperature for 30 min. This was followed by blocking overnight at 4 °C with continuous shaking in 2% BSA and 5% goat serum in PBST. Subsequently, embryos were sequentially incubated with primary and secondary antibodies at 4 °C overnight with continuous shaking, with six washes conducted in between each antibody incubation. The primary antibodies used: rabbit anti-Rasip1 (1:500) [32] and mouse anti-human ZO-1 (Invitrogen, #33-9100, 1:500). The secondary antibodies used: Alexa 568 goat anti-rabbit immunoglobulin G (IgG) (1:1000; Thermo Fisher Scientific, A-11011) and Alexa 488 goat anti-mouse IgG (H + L) (1:1000; Thermo Fisher Scientific, A32723). Following immunostaining, embryos were mounted in 0.75% low-melting-point agarose and imaged using a Leica SP5 microscope equipped with a 40x water immersion objective.

## Results

### Luminal pockets undergo oscillations with local constrictions along junctions

**Fig. 1:**
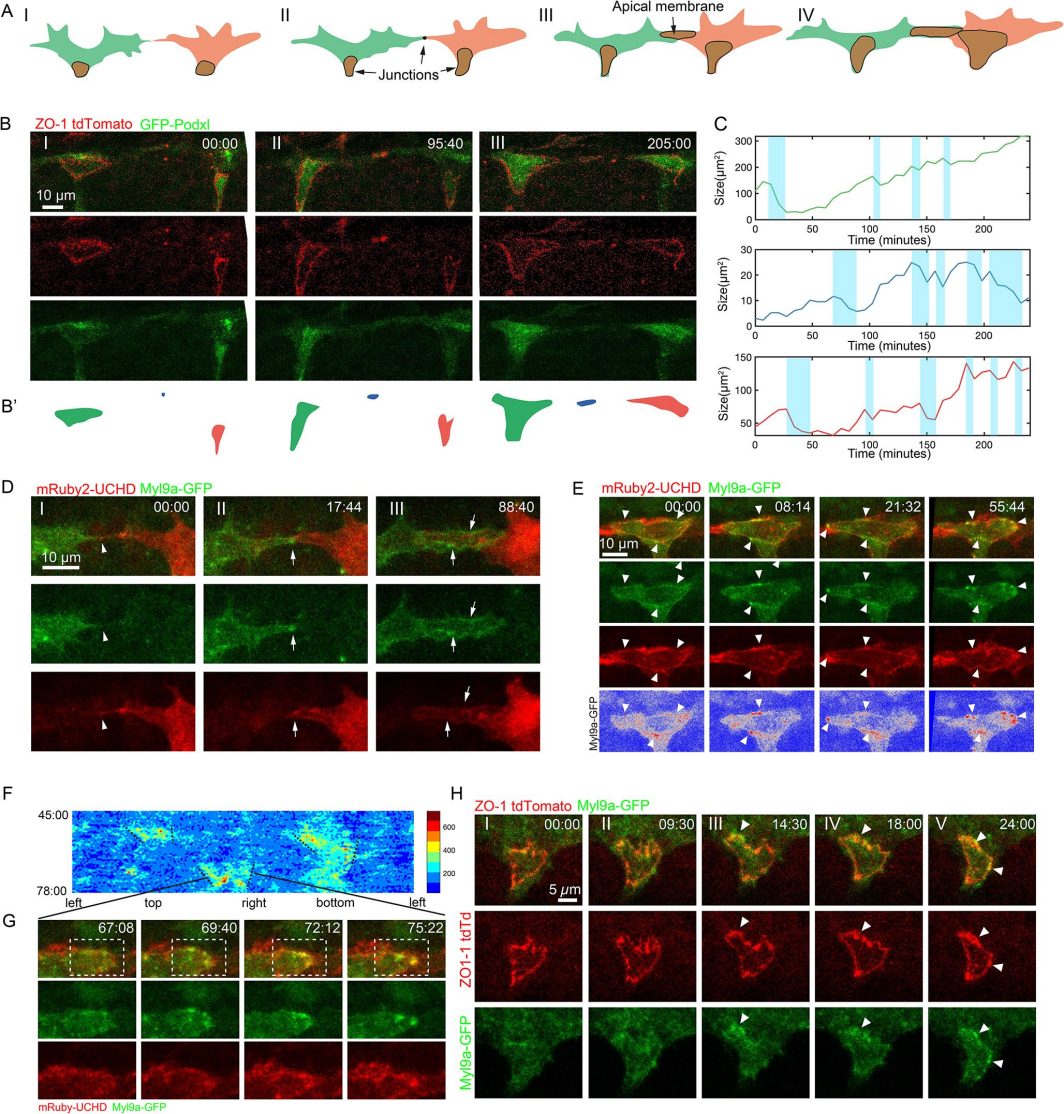
Oscillatory constrictions along junctions maintain the shape of luminal pockets. (**A**) Schematic depicting the anastomosis of two tip cells in the formation of the DLAV. (I) Tip cells establish initial contact with filopodia. (II) Stable cell-cell contact is formed with the deposition of junctional proteins, including Cdh5. (III) At the cell-cell contact site, a luminal pocket is formed, with the apical membrane positioned centrally and encircled by a junctional ring. (IV) Cell rearrangement leads to the expansion of luminal pockets. (**B** and **B**’) Time-lapse imaging of ZO-1 tdTomato and Cdh5-Venus, alongside a corresponding schematic illustrating the formation and expansion of luminal pockets. (**C**) Changes in the size of luminal pockets displayed in (**B**), color-coded to match the regions in (**B**’). (**D**) Sequential imaging capturing the transition from initial filopodia contact to the establishment of well-defined luminal pockets, using mosaically labeled Myl9a-GFP and mRuby2-UCHD. (**E**) Time-lapse visualization of Myl9a-GFP and mRuby2-UCHD, revealing dynamic local enrichment of Myl9a-GFP along junctions across different spatial and temporal scales. White arrowheads indicate the localized enrichment of Myl9a. (**F**) Conversion of Myl9a-GFP signals observed in (**E**) into a 2D representation along junctions, with the x-axis denoting ring positions and the y-axis representing time. Dashed black lines indicate the progressive narrowing of Myl9a-GFP signals during each pulsatile event. (**G**) Time-lapse imaging showcasing localized constriction events as highlighted in (**F**), employing Myl9a-GFP and mRuby2-UCHD labeling. (**H**) Dynamic visualization of Myl9a-GFP and ZO-1 tdTomato illustrating how constrictions facilitate the straightening of junctions over time. White arrowheads indicate the localized enrichment of Myl9a

In zebrafish embryos, continuous lumen formation in the DLAV involves the creation and coalescence of luminal pockets between adjacent cells (Fig. 1A). During this process, initial contact between the tip cells at the top of sprouts is established by filopodial interactions (Fig. 1A(I)). Stable contacts are subsequently established through the deposition of adherens junction (AJ) proteins (Fig. 1A(II)). A *de novo* apical compartment is then formed at the adhesion site, creating a luminal pocket with junctions localized at the periphery (Fig. 1A(III)). The tip-tip luminal pocket expands through cell rearrangement and connects with other luminal pockets formed between tip and stalk cells, facilitating the establishment of vascular patency (Fig. 1A(IV)). By employing ZO-1 tdTomato and GFP-Podxl1 as markers for junctional and apical domains, respectively, we monitored the initiation and enlargement of tip-tip and tip-stalk luminal compartments (Fig. 1B; Video 1). Initially, a significant fraction of GFP-Podxl1 was observed outside the expected apical regions. However, GFP-Podxl1 predominantly relocated to the presumed apical compartments within the junctional rings as the luminal pockets expanded. To further investigate the dynamics of cell-cell contact establishment and luminal pocket expansion, we quantified changes in the size of the luminal compartments within junctional rings over a 4-hour period starting from 30 hpf (Fig. 1C). Both the tip-tip and tip-stalk junctional rings exhibited an overall increase in size. Nonetheless, intermittent decreases (blue regions) in size were noted, suggesting oscillatory behaviours characterized by alternating phases of expansion and constriction.

To investigate the oscillatory behaviors of junctional rings, we utilized mRuby2-UCHD and Myl9a-GFP as markers for the actin-myosin cytoskeleton (Fig. 1D). Mosaic labeling of UCHD and Myl9a in tip cells allowed for the precise distinction between the two cells during anastomosis. Initial contact between the two tip cells was established via filopodia (Fig. 1D(I)). Subsequently, Myl9a enrichment was observed at the stabilized contact site (Fig. 1D(II)). After the formation of the apical compartment, a significant portion of Myl9a localized along the junctional ring encircling the luminal pocket (Fig. 1D(III)). Detailed examination of Myl9a localization revealed its dynamic enrichment along various junctional regions at different time points (Fig. 1E; Video 2). Next, we unrolled the Myl9a distribution along the junctional ring and projected it onto a 2D map, with the x-axis representing relative positions and the y-axis representing time, to assess the dynamics of Myl9a along the junctions over time intervals ranging from 45 to 78 min (Fig. 1F). In line with prior observations, we detected concentrated Myl9a enrichment at specific junctional sites, exhibiting brief temporal pulses. Concurrently, we noted a reduction in the size of these enriched regions within each pulse, indicating localized constrictive events (dotted black lines). Consistent with this analysis, we observed Myl9a enrichment accompanied by localized constrictions at junctions during the pulse (Fig. 1G). Live imaging with ZO-1 indicated that these constrictive events straightened discontinuous or zigzag-shaped junctions following the expansion of luminal pockets (Fig. 1H; Video 3). Taken together, we observed oscillatory constrictions at the endothelial cell-cell junctions, as indicated by multiple junctional and apical reporters. The local accumulation of Myosin, demonstrated by Myl9a reporters, suggests the presence of actomyosin-mediated contractions at these junctions, hereafter referred to as junctional contractility.

#### *heg1* and *krit1* mutants display contorted junctions and fragmented apical domains

**Fig. 2:**
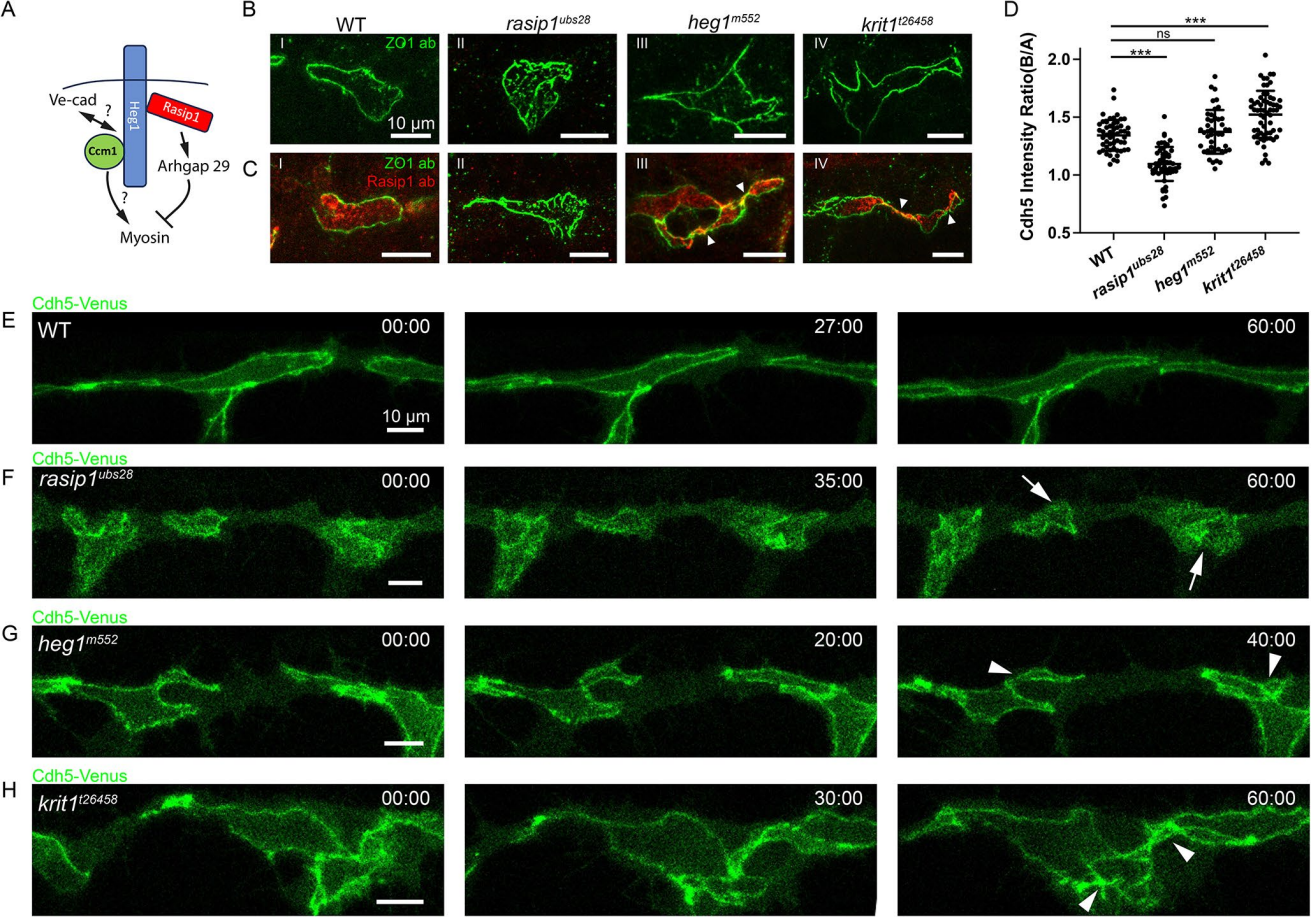
Zigzag and contorted junctions occur in *heg1* and *krit1* mutants. (**A**) Diagram showing interactions between Rasip1, Heg1, and Krit1. (**B** and **C**) Immunofluorescence staining of ZO-1 alone (**B**) or in combination with Rasip1 (**C**) in wild-type embryos, *rasip1*, *heg1*, and *krit1* mutants at 32 hpf. White arrowheads indicate the contorted junctions in *heg1* mutants and *krit1* mutants. (**D**) Quantification of the Cdh5 boundary-to-apical ratio in wild-type embryos (*n* = 53), *rasip1* mutants (*n* = 55), *krit1* mutants (*n* = 62), and *heg1* mutants (*n* = 43), presented as mean ± SD. (**E**-**H**) Time-lapse imaging of Cdh5-Venus in wild-type embryos (**E**), *rasip1* mutants (**F**), *heg1* mutants (**G**) and *krit1* mutants (**H**) at DLAV starting from 33 hpf. White arrows indicate the poorly defined junctions in *rasip1* mutants. White arrowheads indicate the contorted junctions in *heg1* mutants and *krit1* mutants

Rasip1, Heg1, and Krit1 are involved in the regulation of Rho GTPases, which are implicated in regulating endothelial contractility. Rasip1 and Krit1 bind to Heg1, a transmembrane protein, at different binding sites on the cytoplasmic domain of Heg1 (Fig. 2A) [19, 20]. We subsequently investigated the roles of Rasip1, Heg1, and Krit1 in the regulation of cell-cell junctions through analyses of *rasip1*^*ubs28*^, *heg1*^*m552*^, and *krit1*^*t26458*^ mutants [13, 32, 33]. Immunostaining of ZO-1 revealed that *heg1* and *krit1* mutants exhibited zigzag or ectopically contorted junctions, in contrast to the ring-shaped junctions observed in wild-type embryos (Fig. 2B and C). These observed phenotypes sharply contrasted with those of *rasip1* mutants, which displayed ectopic junctional materials in the apical compartments. In contrast, the apical compartments in *heg1* and *krit1* mutants were largely devoid of junctional complexes (Fig. 2B and D). In wild-type embryos, Rasip1 was largely restricted to the presumed apical domains inside the junctional rings (Fig. 2C). Similarly, Rasip1 was found at the cell-cell interfaces in *heg1* and *krit1* mutants, indicating that its apical localization is largely independent of Heg1 and Krit1 (Fig. 2C). However, in *heg1* and *krit1* mutants, the apical domains exhibited fragmented distributions within the loops of contorted junctions, which were connected by bottlenecked regions (Fig. 2C). Thus, the contorted junctions observed in *heg1* and *krit1* mutants appeared to disrupt the integrity of the apical domains. We observed similar phenotypes using the Cdh5-Venus live reporter. In wild-type embryos, the junctional rings approached each other, with each ring straightened along the direction of the vessel (Fig. 2E). In contrast, the boundaries of the presumed apical compartments in *rasip1* mutants appeared fuzzy and discontinuous, with widespread distribution of Cdh5-Venus (Fig. 2F). Although the junctions in *heg1* and *krit1* mutants appeared sharp, they were not straightened, and the ectopic twisting persisted, as seen in live imaging (Fig. 2G and H).

#### Aberrant control of cell-cell interface shape in *heg1* and *krit1* mutants

**Fig. 3:**
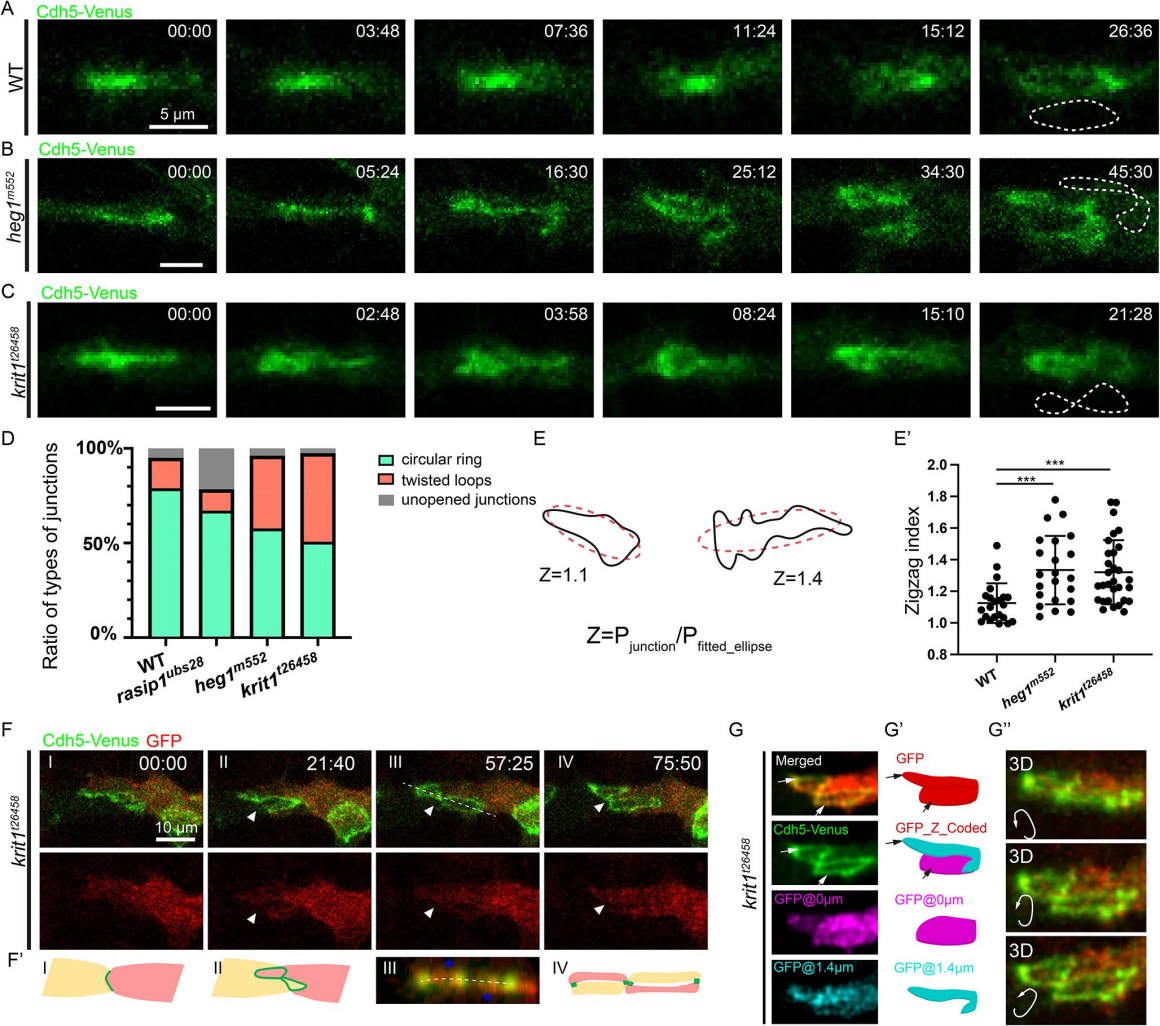
Ectopic shape control of endothelial cell-cell interfaces in *heg1* and *krit1* mutants. (**A**-**C**) Time-lapse imaging of Cdh5-Venus depicting the transition from junctional patches to circular rings in wild-type embryos (**A**) and “∞”-shaped junctions in *heg1* mutants (**B**) and *krit1* mutants (**C**). (**D**) Distribution of different junctional types in wild-type embryos (n = 57), *rasip1* (n = 73), *heg1* (n = 71), and *krit1* (n = 69) mutants. (**E** and **E**’) Definition and quantification of the zigzag index in wild-type embryos (*n* = 22), *heg1* (*n* = 22) and *krit1* (*n* = 30) mutants. (**F** and **F**’) Time-lapse imaging and corresponding schematic illustrating “∞”-shaped junction formation in *krit1* mutants with Cdh5-Venus and mosaically expressed GFP. Cross-sectional views along dashed lines in F(III) are shown in F’(III) and F’(IV), with blue asterisks indicating GFP-positive cells and white arrowheads highlighting crossing points between loops. (**G**) Visualization of “∞”-shaped junctions at various Z positions with GFP labeling in a single cell. GFP fluorescence at the upper loop was detected at 1.4 μm (blue), contrasting with the lower loop where GFP was visible at 0 μm (red), indicating differential Z-plane localization of the two loops. (**G**’) 3D diagrams of cell shape at contorted cell-cell interfaces, with color coding based on Z positions. (**G**’’﻿) 3D reconstructions of (**G**) viewed from different angles. *** *P* < 0.001, ns *P* > 0.05 (t-test)

To determine whether the contorted shape of junctions reflects extended/twisted cell-cell interfaces or an increased number of interconnections between cells, we performed live imaging on the initial adhesion sites and followed cell rearrangements (Fig. 3A-C). In wild-type embryos, most initial adhesion sites between two cells opened into circular junctional rings (Fig. 3A and D). In contrast, a significant proportion of single adhesion sites transitioned directly into “∞”-shaped junctions with two interconnected loops in *heg1* (27/71) and *krit1* (32/69) mutants, while the remaining adhesion sites transformed into more or less circular junctional rings (Fig. 3B-D). Thus, the ectopic “∞”-shaped junctions consisted of a continuous cell-cell interface between two adjacent cells, rather than multiple interconnected rings formed by three or more cells. To elucidate alterations in the shape of cell-cell interfaces in *heg1* and *krit1* mutants, we first quantified the shape of the one-loop junctional rings in the two mutants and compared them to those in wild-type embryos. The zigzag index (Z) was defined as the ratio of the perimeter of the junctional ring (P_junction) to the perimeter of the fitted ellipse (P_fitted_ellipse) (Fig. 3E). Our quantifications revealed an increased zigzag index in *heg1* and *krit1* mutants, even in the relatively normal portions of junctions (Fig. 3E’). To understand the geometry of these “∞”-shaped cell-cell interfaces and the shape of ECs, we performed live imaging with mosaic labeling of ECs in the DLAV (Fig. 3F; Video 4). Reconstructed orthogonal sections of the “∞”-shaped junctions showed that the labeled tip cell was positioned above the other cell in one loop, but below it in the other loop (Fig. 3F(III)). 3D reconstruction of “∞”-shaped junctions demonstrated that the two loops were in different z-planes, with an intersecting point where one junction lay on top of the other (Fig. 3G-G’’; Video 5). Thus, instead of being positioned opposite each other, the two tip cells intersected and lay on both the top and bottom sides of each other, leading to the formation of two interconnected but flipped interfaces.

### Heg1 and Krit1 are required for maintaining junctional contractility

**Fig. 4:**
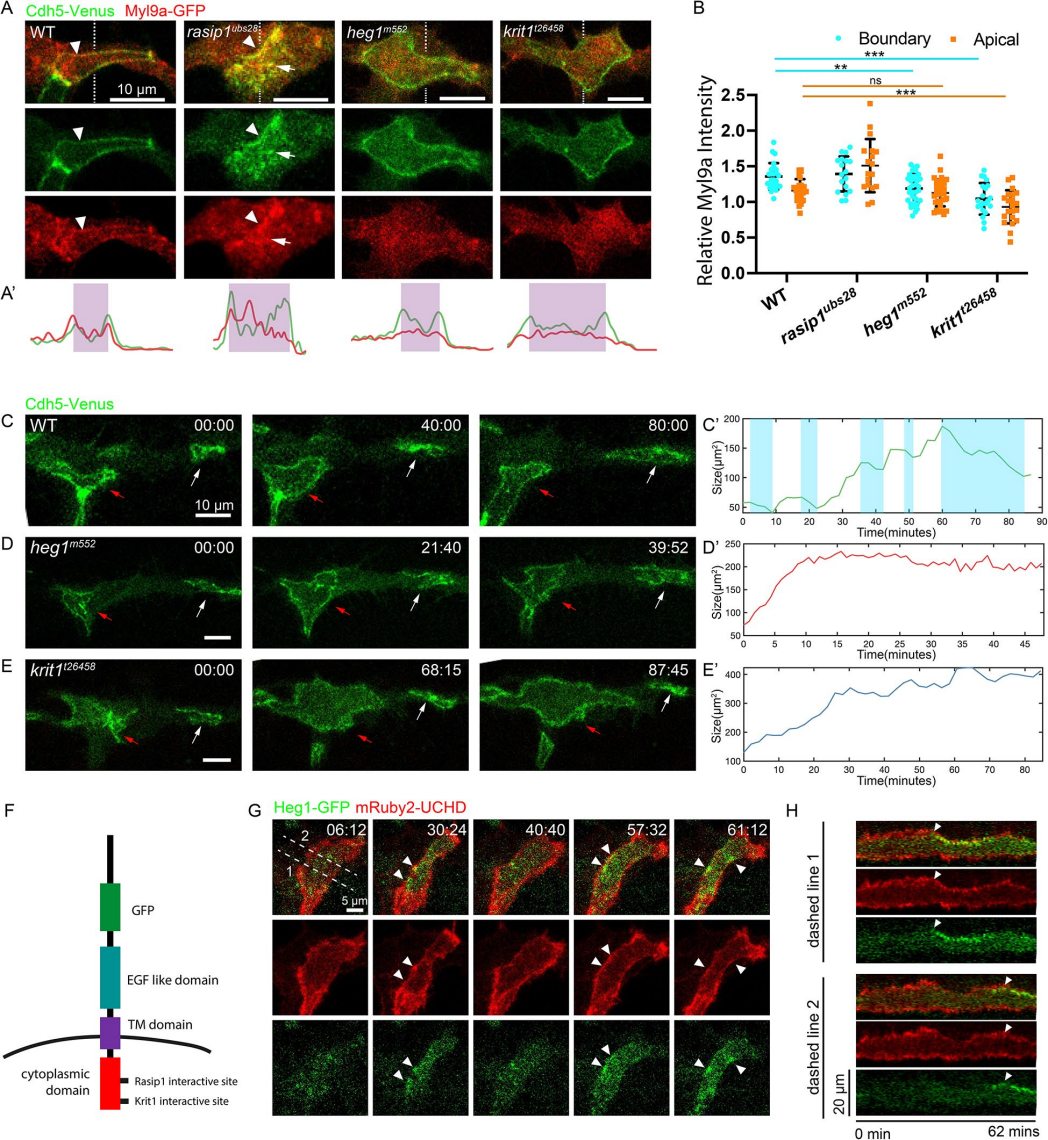
Heg1 and Krit1 control contractility along junctions. (**A**) Myl9a-GFP and Cdh5-Venus in wild-type embryos, *rasip1*, *heg1*, and *krit1* mutants at 32 hpf. White arrowheads indicate junctional Myl9a, while white arrow point to apical Myl9a in *rasip1* mutants. (**A**’) Quantification of Myl9a-GFP and Cdh5-Venus intensities along the dashed lines shown in (**A**). (**B**) Normalized Myl9a intensities at junctional regions and apical compartments in wild-type embryos (*n* = 25), *rasip1* mutants (*n* = 18), *heg1* mutants (*n* = 34), and *krit1* mutants (*n* = 21), presented as mean ± SD. Myl9a-GFP intensity is standardized using the mean signal level from the entire cell. (**C**-**E**) Time-lapse imaging of Cdh5-Venus in wild-type embryos (**C**), *heg1* mutants (**D**), and *krit1* mutants (**E**), illustrating the expansion of luminal pockets between stalk and tip cells and the establishment of luminal pockets between tip cells. Stalk-tip luminal pockets appear zigzag (red arrows), while newly established tip-tip luminal pockets exhibit “∞”-shaped structures (white arrows) in *heg1* (**D**) and *krit1* mutants (**E**). (**C**’-**E**’) Changes in the size of stalk-tip luminal pockets in (**C**-**E**). (**F**) Illustration of recombined Heg1-GFP with GFP inserted in the middle of the extracellular domain. (**G**) Visualization of Heg1-GFP and mRuby2-UCHD in wild-type embryos at 32 hpf, showing the local recruitment of Heg1 to junctions during constrictions. White arrowheads highlight locally enriched Heg1-GFP. (**H**) Kymographs generated along the dashed lines in (**G**), demonstrating the recruitment of Heg1-GFP during local constrictions. White arrowheads indicate the recruitment of Heg1-GFP at the onset of constrictions

The zigzag and twisted junctions observed in *heg1* or *krit1* mutants suggested that the actomyosin contractility along the junctions might be compromised. To test this hypothesis, we utilized Myl9a-GFP as a reporter of actomyosin activity in *rasip1*, *heg1*, and *krit1* mutants, as we did in wild-type embryos (Fig. 4A and B). In wild-type embryos, Myl9a predominantly localized along the junctions. In *rasip1* mutants, consistent with the role of Rasip1 as an inhibitor of actomyosin contractility at the apical compartment, Myl9a-GFP was ectopically enriched within the apical compartments. Our prior investigation revealed that heightened myosin activity at the apical compartments (hereafter referred to as apical contractility) pulled junctional complexes from the junctions into the apical compartments, thus destabilizing cell-cell junctions [31]. In contrast, there was no obvious apical or junctional enrichment of Myl9a-GFP in *heg1* and *krit1* mutants, suggesting a loss of junctional contractility as we hypothesized (Fig. 4A and B). Live imaging of *heg1* and *krit1* mutants revealed the rapid expansion of luminal pockets between the tip and stalk cells, characterized by zigzag boundaries, and the formation of “∞”-shaped junctions between tip cells (Fig. 4C-E; Video 6–8). Quantitative analysis showed a lack of oscillatory constrictions in *heg1* and *krit1* mutants, in sharp contrast to wild-type embryos (Fig. 4C’-E’). Thus, while Rasip1 inhibits actomyosin contractility within the apical compartments and along the junctions, Heg1 and Krit1 maintain actomyosin contractility along the junctions. These observations align with the previously identified role of Krit1 in recruiting Rock2 to VE-cadherin–β-catenin complexes [18].

To investigate the involvement of Heg1 and Krit1 in oscillatory local constrictions at the protein level, we engineered a Heg1 reporter line with GFP fused to its extracellular domain [*Tg(fli: gal4; UAS: Heg1-GFP*^*61*^] (Fig. 4F). Notably, Heg1-GFP exhibited strong intermittent enrichment at local regions of the junctions, closely associated with instances of local constrictions (Fig. 4G; Video 9). Analysis using kymographs constructed from Heg1-GFP and mRuby2-UCHD signals indicated that Heg1-GFP was recruited to the constricting junctions at the onset of the constriction phase, persisting until the local region expanded once more (Fig. 4H). Similarly, Rasip1-Scarlet-I, akin to Heg1-GFP, exhibited dynamic recruitment to the sites of junctional constriction (Fig. S1A and S1B). Another potential interactive partner, Radil2a (Radil-B), showed similar dynamic shuttling between the apical compartments and junctions during local constrictive events [*Tg(fli: gal4; UAS: GFP-Radil2a)*^*ubs62*^] [19] (Fig. S1C and S1D). Combined with the functional studies, these observations indicate that Heg1 and its interactive partners are dynamically recruited to junctions and are required for regulating local actomyosin contractility.

### Essential junctional contractility shapes cell-cell interfaces and apical integrity

**Fig. 5:**
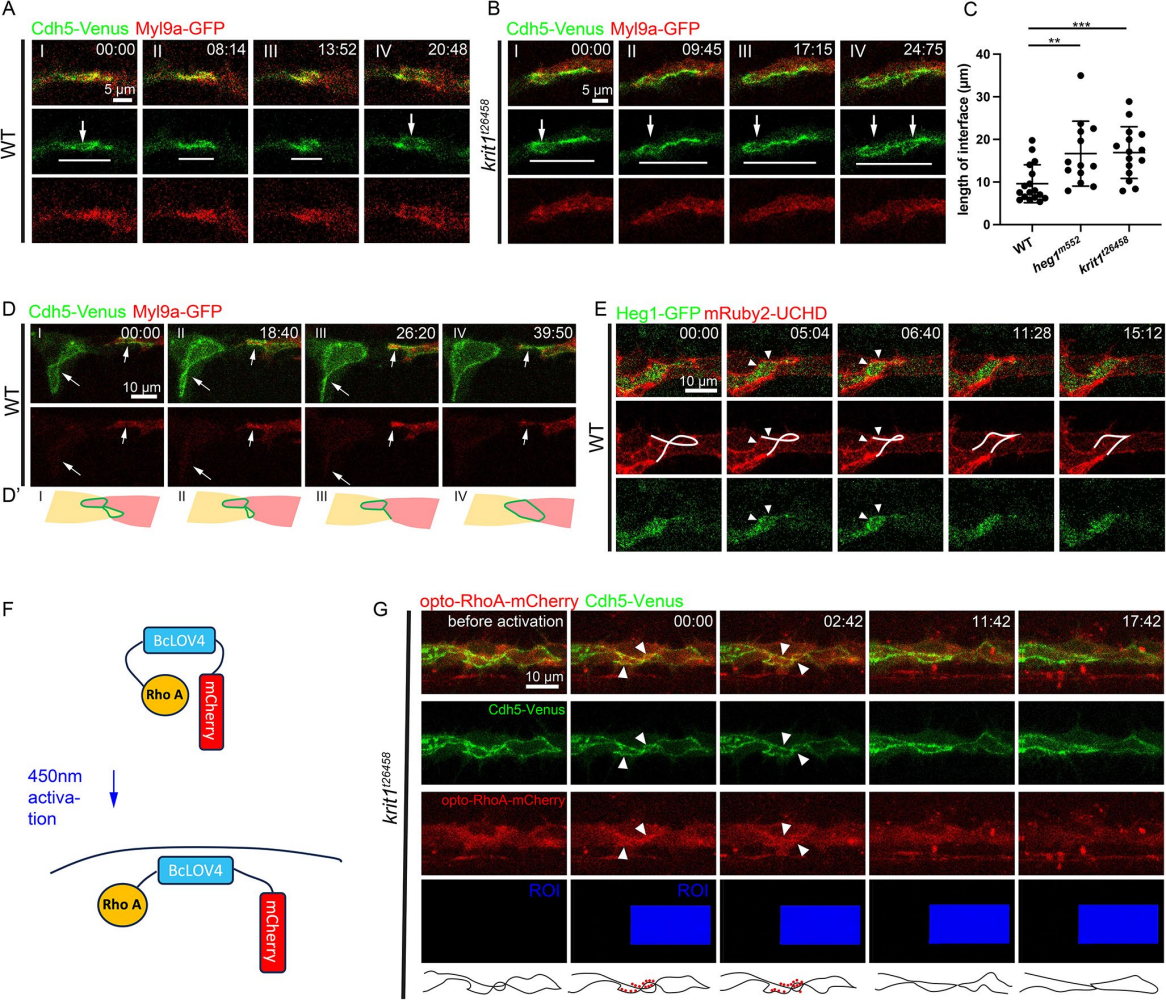
Essential junctional contractility refines cell-cell interactions. (**A** and **B**) Time-lapse imaging of Cdh5-Venus and Myl9a-GFP showing the transition from junctional patches to circular rings in wild-type embryos (**A**) and “∞”-shaped junctions in *krit1* mutants (**B**). White arrows denote nascent or stabilized junctional rings, while white lines indicate the linear junctional interface before it opens into junctional rings. (**C**) Quantification of the length of linear junctional interfaces between tip cells prior to their conversion into junctional rings in wild-type embryos (n = 17), *heg1* mutants (n = 13), and *krit1* mutants (n = 15). (**D** and **D**’) Time-lapse imaging and corresponding diagrams depicting the untying of “∞” shape junctions in wild-type embryos with Cdh5-Venus and mosaically expressed Myl9a-GFP. White arrows highlight the resolution of loops. (**E**) Time-lapse imaging of Heg1-GFP and mRuby2-UCHD, revealing the local enrichment of Heg1-GFP during the retraction of junctional loops to disentangle “∞”-shaped junctions in wild-type embryos. (**F**) Schematic diagram of the single-component opto-RhoA system. Blue light activation induces dynamic membrane recruitment of cytosol-sequestered RhoA-BcLOV4-mCherry. (**G**) Activation of opto-RhoA straightens twisted junctions in *krit1* mutants and corresponding diagrams. White arrowheads indicate the opto-RhoA clusters enriched along junctions after activation. *** *P* < 0.001, ** *P* < 0.01 (t-test)

To explore the link between junctional contractility and the regulation of cell shape and apical connectivity, we investigated the establishment of cell-cell interfaces and myosin activities during anastomosis in wild-type embryos and *krit1* mutants. In wild-type embryos, the initial adhesion site underwent dramatic remodelling, with the transient establishment and subsequent collapse of immature junctional rings before the formation of stable luminal pockets (Fig. 5A). The immature junctional ring underwent constriction and collapse, accompanied by increased Myl9a enrichment, thereby further narrowing the interface between two tip cells (Fig. 5A(II and III)). Immature junctional rings were also observed in *krit1* mutants within the partially opened cell-cell interfaces between two tip cells (Fig. 5A(I) and 5B(I)). However, in contrast to wild-type embryos, the immature junctional rings persisted in *krit1* mutants (Fig. 5B(II)). In the absence of Myl9a enrichment and oscillatory constrictions, the linear cell-cell interface between two tip cells continuously extended (Fig. 5B(III), Fig. 3B and C). New junctional loops formed at the extended linear interface, likely on the reverse side of the old loop, resulting in “∞”-shaped junctions in *krit1* mutants (Fig. 5B(IV)). We quantified the length of the linear interface between two tip cells before its transformation into junctional rings (white lines in Fig. 5A and B). Our analysis revealed that *heg1* and *krit1* mutants displayed significantly longer linear interfaces, possibly due to the absence of effective junctional contractility (Fig. 5C). This prolonged interface increased the likelihood of forming multiple junctional loops on both sides, leading to distorted junctions and fragmented apical domains.

Intriguingly, contorted junctions were also observed in a fraction of wild-type embryos (9/57) (Fig. 5D; Video 10). In wild-type embryos, most twisted rings (7/9) were disentangled by the retraction of one of the two loops. This process resulted in the formation of continuous luminal pockets encircled by circular junctions. Concurrently, we observed a local enrichment of Myl9a and Heg1 along cell-cell junctions close to the constricting site during the retraction (Fig. 5D and E). In contrast, the contorted junctions persisted (Fig. 2G and H), and even relatively normal circular rings twisted into contorted shapes in both *heg1* and *krit1* mutants (Fig. S2A-C). Hence, we hypothesized that junctional contractility is required to straighten junctions, thereby controlling the shape of cell-cell interfaces and ensuring proper apical integrity in ECs during anastomosis. To test this hypothesis, we applied opto-RhoA at the twisted junctions in *krit1* mutants to induce local activation of RhoA signaling [35] (Fig. 5F). After continuous 450 nm illumination at the region of interest (ROI), the coiled junctions became straightened and untied with the recruitment of RhoA-BcLOV4-mCherry clusters to the junctions (Fig. 5G; Video 11). Hence, sufficient junctional contractility is required for the straightening of junctions and the removal of excessive cell-cell interfaces, thereby preserving the appropriate shape of cell-cell interfaces and apical integrity.

#### *heg1* and *krit1* mutants fail to establish stable cell-cell contact and interconnected luminal space

**Fig. 6:**
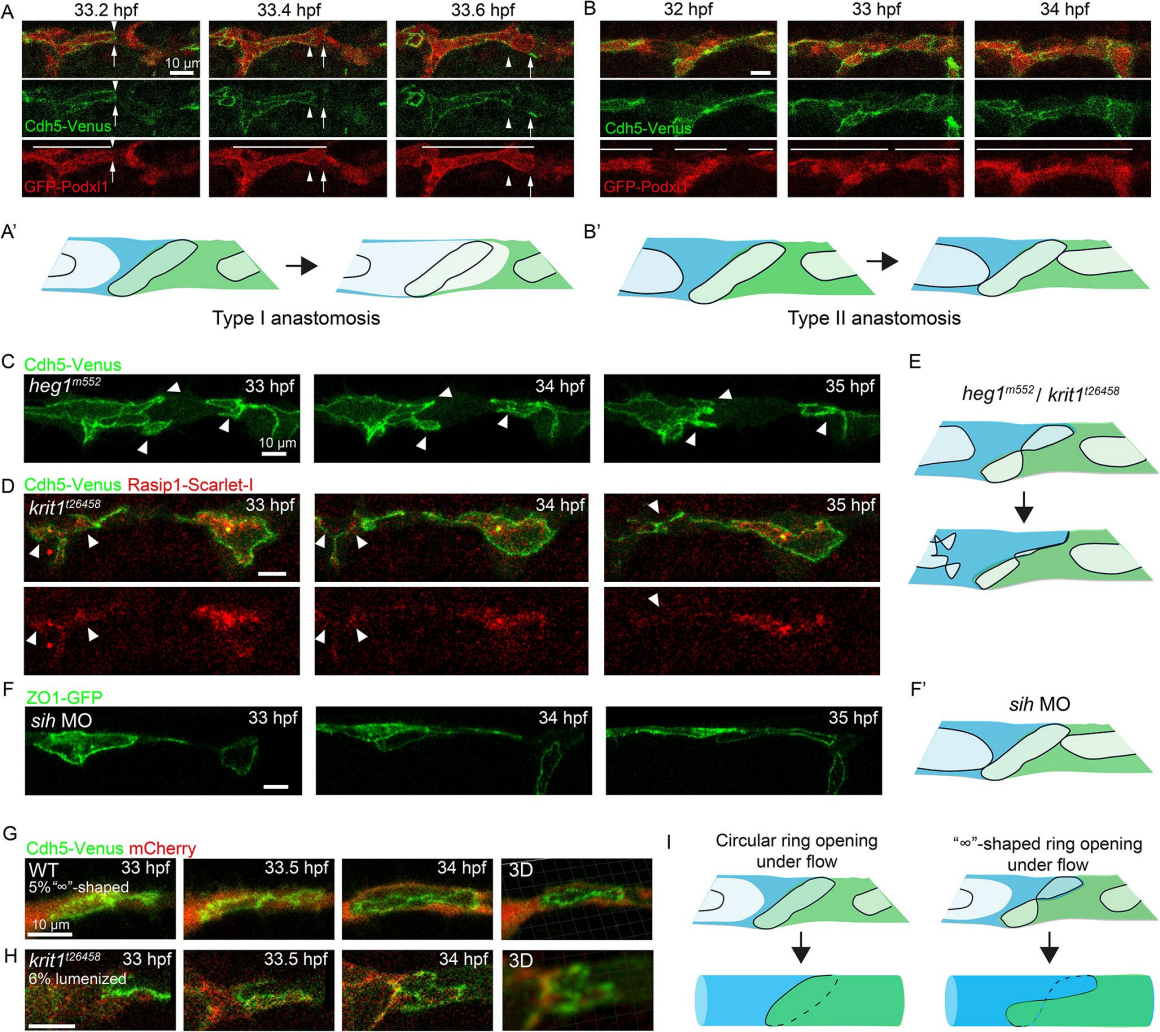
Actomyosin contractile forces and blood flow shape endothelial cell-cell interactions. (**A** and **B**) Time-lapse imaging of Cdh5-Venus and GFP-Podxl1 illustrating lumenization in type I and type II anastomoses in wild-type embryos. In type I anastomosis, the apical membrane invaginates due to blood pressure (white arrows). In type II anastomosis, luminal pockets expand through cell rearrangement, resulting in lumen coalescence (white lines). White arrowheads mark the distal end of junctional rings, while white arrows indicate the lumenized vessel. White lines label luminal spaces. (**A**’ and **B**’) Schematic diagrams depicting the establishment of continuous luminal space under type I and type II anastomoses. (**C**-**E**) Time-lapse imaging of Cdh5-Venus showing the collapse of contorted junctions in *heg1* (**C**) and *krit1* (**D**) mutants, with a corresponding diagram (**E**). White arrowheads denote junctions undergoing collapse. (**F**) Time-lapse imaging of ZO1-GFP in *sih* morphants at the same stage, with corresponding diagram (**F**’). (**G** and **H**) Lumenization at DLAV inflated “∞”-shaped junctions into circular junctions in perfused tubes as seen in wild-type embryos (2/45 with “∞”-shaped junctions before lumenization) (**G**) and *krit1* mutants (2/33 with transient lumenization) (**H**). (**I**) Graphic diagrams illustrating blood pressure-driven lumenization through circular junctions or “∞”-shaped junctions

So far, our findings reveal that the excessive cell-cell interfaces and fragmented apical domains in *heg1* and *krit1* mutants are due to a lack of junctional contractility. These phenotypes were observed and studied in ECs during the anastomosis of the DLAV at around 32 hpf before the establishment of blood flow. We now ask how the proper control of cell-cell interfaces and apical integrity are relevant to the formation of perfused blood vessels. In wild-type embryos, the formation of interconnected luminal space within the vasculature involves two different cellular mechanisms referred to as type I and type II anastomosis [7, 30, 36]. In type I anastomosis, blood pressure pushes the luminal space from one cell to its connecting neighbour. The apical membrane on the expanding lumen fuses with the *de novo* inserted apical membrane within the luminal pocket, thereby generating a continuous intracellular lumen (Fig. 6A and A’). In contrast, type II anastomosis can occur in the absence of blood pressure. The connection of luminal space is driven by the expansion of luminal pockets via the rearrangement of cells. The *de novo* inserted luminal pockets eventually connect with each other, leading to lumen coalescence and the formation of a multicellular tube (Fig. 6B and B’).

Zebrafish *heg1* and *krit1* mutants form massively dilated hearts and fail to establish blood flow [13, 33, 37]. Consequently, type I anastomosis is unlikely to occur in *heg1* and *krit1* mutants due to the absence of blood pressure. The contorted junctions in *heg1* and *krit1* mutants further twisted and eventually collapsed into dense junctional patches or linear structures (Fig. 6C-E; Video 12 and 13). The collapsed junctions failed to connect with other junctions, thereby hindering type II anastomosis and preventing the formation of interconnected luminal spaces. The fragmented apical domains collapsed with the surrounding junctions in *krit1* mutants (Fig. 6D and E). These observations suggest that, without effective straightening and retraction, the excessive cell-cell interfaces and fragmented apical domains in both mutants are unstable and prone to collapse. To test whether blood flow is required for the straightening of junctions, we injected antisense morpholinos against cardiac troponin (*silent heart*, *sih*, *tnnt2a*) and found that junctional rings were straightened along the vessels in the absence of blood flow (Fig. 6F and F’). Thus, the formation of interconnected luminal space in *heg1* and *krit1* mutants was impeded by the absence of blood flow and inadequate cell rearrangement. The insufficient cell rearrangements are independent of blood flow but are subject to the excessive and unstable cell-cell contacts in the absence of junctional contractility in both mutants.

### Actomyosin contractile forces and blood flow together shape endothelial cell-cell interactions

There is curiosity regarding the fate of the contorted junctions if blood flow were to resume, as a previous study has shown that blood flow suppresses vascular anomalies in zebrafish *krit1* mutants [37]. In fact, a very small fraction (2/45) of wild-type embryos displayed “∞”-shaped junctions before lumenization (Fig. 6G; Video 14), and a very small portion (2/33) of *krit1* mutants displayed transient lumenization at the DLAV (Fig. 6H; Video 15), providing opportunities to explore type I anastomosis with contorted junctions. Under blood pressure, the “∞”-shaped junctions were inflated, with the intersecting points of the two loops detaching and moving to opposite sides of the perfused compartment (Fig. 6I). The inflated “∞”-shaped junctions became circular in the tube, as the disentangling occurred in 3D, in contrast to the 2D disentangling with loop retraction (Fig. 6I). Thus, blood flow facilitates the formation of continuous luminal spaces through contorted junctions. Hemodynamic forces appear to render contorted junctions less hazardous, potentially by opening luminal spaces through contorted junctions and restoring tension.

## Discussion

The mechanism by which the loss of CCM protein signalling leads to vascular malformations remains largely unknown. In this study, we combined advanced live imaging and genetic analysis to investigate the diverse roles of the Krit1-Heg1-Rasip1 complex in the patterning of a functional vasculature. In *heg1* and *krit1* mutants, we observed distorted and excessive junctions, indicating a failure in regulating the shape of endothelial cell-cell interfaces. The presence of zigzag and contorted junctions, coupled with the absence of constriction phases and the loss of Myl9a enrichment along junctions, collectively indicate the essential roles of Heg1 and Krit1 in initiating oscillatory constrictions along junctions. Activation of actomyosin contractility with opto-RhoA straightened and untied the twisted junctions in *krit1* mutants, supporting the essential role of actomyosin contractility in the regulation of proper endothelial cell-cell interfaces. In the absence of effective junctional straightening and retraction due to the loss of junctional contractility, the contorted cell-cell interfaces fragmented apical domains in *heg1* and *krit1* mutants. The contorted cell-cell interfaces, along with the fragmented apical domains, hindered proper cell rearrangement along the vessel, thereby impeding continuous lumen formation in type II anastomosis. On the protein level, we revealed the dynamic recruitment of Heg1 and its interactive partners to local constriction sites by employing engineered reporters of Heg1, Rasip1, and RadilB. Above all, our study revealed that Heg1 and Krit1 are dynamically recruited to local junctions and are required for generating oscillatory contractile forces along junctions, thus regulating the shape of cell-cell interfaces and continuous luminal space formation during the development of a functional vasculature.

Our observations starkly contrast with prior studies indicating increased actomyosin contractility due to heightened RhoA and ROCK activity in *heg1* and *krit1* mutants [14–17]. This disparity in interpretation may stem from differing cellular focuses, with previous studies predominantly examining cell-extracellular matrix adhesions and stress fiber dynamics, while our investigation centers on cell-cell junctions. Notably, another study showed that Krit1 regulates ROCK1 and ROCK2 differentially [18]. Krit1 recruits ROCK2 to cell-cell junctions but inhibits ROCK1-dependent actin stress fibres, thereby ensuring a proper balance between cell-extracellular matrix adhesions and cell-cell junctions.

### Oscillatory contractions and elongations shape endothelial cell-cell interface and lumen

**Fig. 7:**
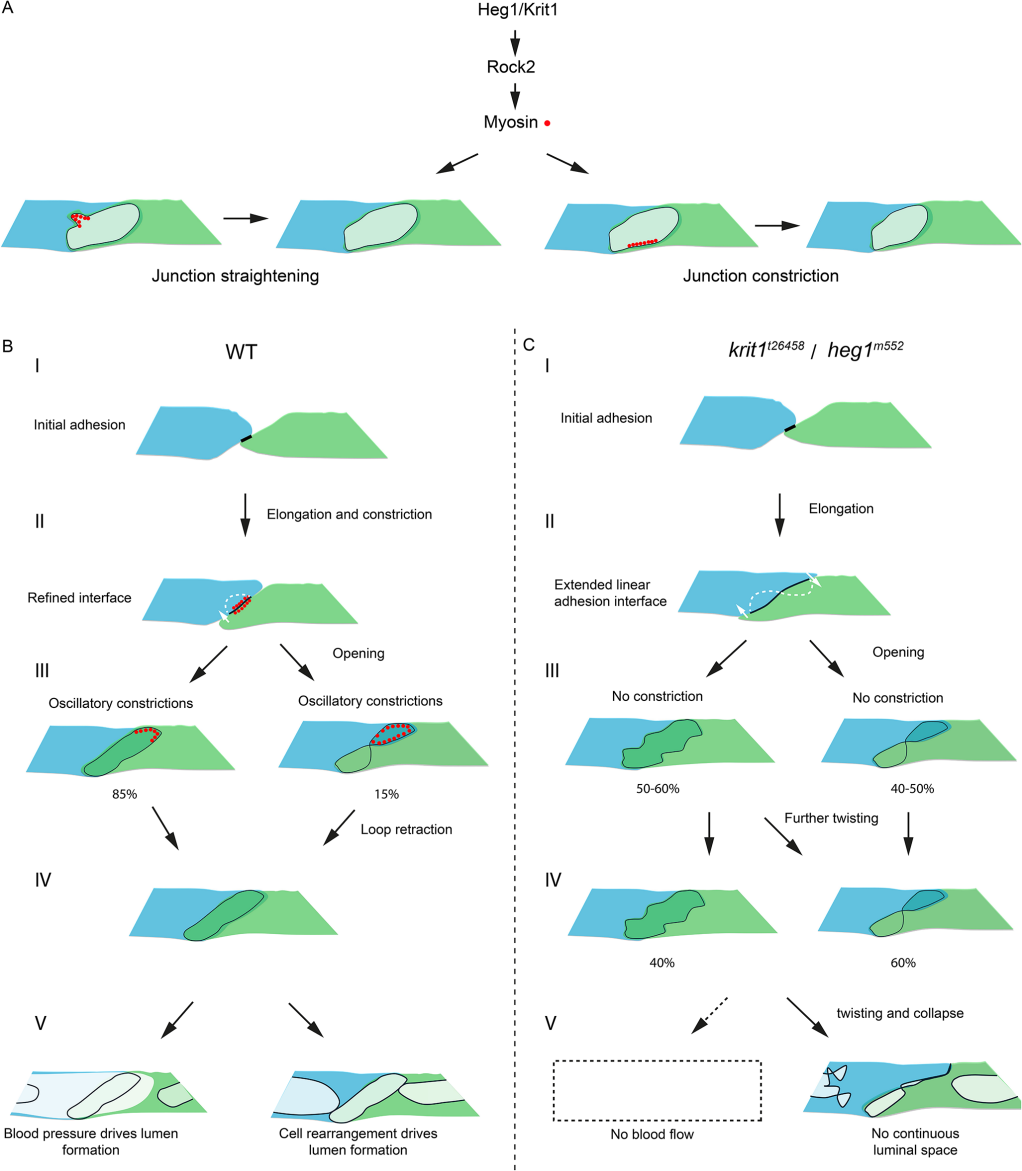
Essential junctional contractility underlies proper cell-cell interactions regulated by Heg1 and Krit1. (**A**) Graphic diagrams illustrating oscillatory actomyosin constrictions (red dots) occur at various locations of endothelial cell-cell interfaces, which straighten zigzag-shaped or discontinuous junctions and regulate the size of luminal pockets. (**B** and **C**) Graphic diagrams demonstrating the roles of Heg1 and Krit1 in vascular patterning and lumen formation. Tip cells establish initial contact with each other (**B**(I) and **C**(I)). Junctional contractility (red dots) narrows the linear cell-cell interfaces in wild-type embryos (**B**(II)). Longer cell-cell interfaces occur in *heg1* and *krit1* mutants in the absence of junctional contractility, increasing the likelihood of “∞”-shaped junction formation at both sides of a cell (white dashed line) (**C**(II)). After the opening of linear cell-cell interfaces into luminal pockets, oscillatory junctional contractility (red dots) straightens junctions and disentangles contorted junctions by retracting loops, reducing ectopic twists in wild-type embryos (**B**(III and IV)). In contrast, *heg1* and *krit1* mutants exhibit persistent or worsened zigzag-shaped or “∞”-shaped junctions, with increased twisting in the absence of essential junctional contractility (**C**(III and IV)). Blood pressure or cell rearrangement drives continuous lumen formation in wild-type embryos (**B**(V)). In contrast, the luminal pockets further twist or collapse in *heg1* and *krit1* mutants, leading to discontinuous luminal space (**C**(V))

During anastomosis, luminal pockets emerge at adhesion sites between tip cells, with the apical membrane inserted into the central region, while the initial adhesion sites transform into junctional rings that delineate the boundaries of the luminal pockets [7, 30, 36]. Following cell rearrangements, the individual luminal pockets expand and connect with each other, leading to the formation of a continuous luminal space. In this study, we describe the oscillatory behaviors of luminal pockets, alternating between expansion and constriction phases, accompanied by pulsed elongation and constriction of junctions. Constrictions occur locally along the junctional rings, characterized by the enrichment of myosin. These localized constrictions facilitate the straightening of discontinuous or zigzag-shaped junctions, thereby regulating the shape of luminal pockets following the expansion phase (Fig. 7A). Our findings suggest that alternating lumen expansion and constriction contribute to the expansion of luminal space while maintaining the proper shape of cell-cell interfaces and apical integrity.

In this study, we identified oscillatory myosin to be localized linearly along specific regions of junctions. The recruitment of myosin at these local junctions heavily relies on Heg1 and Krit1. However, a significant proportion of myosin was also found distally but closely associated with elongating junctions in both wild-type embryos and *heg1* and *krit1* mutants, occurring prior to the expansion of junctional rings (Fig. S3). This observation aligns with the concept of junction-based lamellipodia (JBL) driving stepwise junction elongation at the distal ends of junctions [29]. Thus, it appears that myosin localizes to distinct cellular compartments with different functions during *de novo* lumen formation. Junctional myosin straightens junctions and maintains the proper shape of cell-cell interfaces, while distal myosin promotes junction elongation at the lamellipodia, with the latter being independent of Heg1 and Krit1.

## Redundant mechanisms for proper cell-cell interface and lumen formation

Multiple redundant mechanisms regulate proper cell-cell interactions to ensure robust lumen formation throughout anastomosis. First, following initial tip cell contact (Fig. 7B(I)), actomyosin-mediated contractions are activated at the initial cell-cell adhesion sites, leading to the narrowing of linear cell-cell interfaces before the formation of mature *de novo* luminal pockets in wild-type embryos (Fig. 7B(II and III)). In contrast, prolonged linear cell-cell interfaces occur in *heg1* and *krit1* mutants, leading to the formation of “∞”-shaped junctions with flipped interfaces, resulting in contorted junctions and fragmented apical membranes (Fig. 7C(I-III)). Second, in wild-type embryos, oscillatory contractions disentangle contorted junctions by retracting loops and reducing ectopic twists (Fig. 7B(III and IV)). Circular junctional rings then interconnect, leading to lumen coalescence (Fig. 7B(V)). In contrast, in *heg1* and *krit1* mutants, contorted junctions persist or deteriorate (Fig. 7C(III and IV)). These excessive cell-cell junctions become unstable, collapsing into dense patches or linear structures, which hinder continuous lumen formation (Fig. 7C(V)). Finally, blood pressure can drive continuous lumen formation despite contorted junctions, inflating “∞”-shaped junctions into circular ones (Fig. 6I). Nevertheless, zebrafish *heg1* and *krit1* mutants, with massively dilated hearts and impaired blood flow, fail to establish continuous lumens [13, 33, 36]. Restoration of blood flow suppresses vascular anomalies, including hyperplastic expansion in zebrafish *krit1* mutants [37]. The extent to which deficient junctional contractility and improper cell-cell interfaces contribute to impaired blood flow in *heg1* and *krit1* mutants remains unclear. Further investigations are warranted to elucidate the role of oscillatory constrictions in endocardial cells in establishing blood flow.

### Complementary roles of Rasip1 and Krit1 in stabilizing cell-cell junctions

Both Rasip1 and Krit1 are adaptor proteins downstream of Rap1, a key signaling molecule in both vasculogenesis and angiogenesis [38]. The Rap1–KRIT1–HEG1–RASIP1 complex plays a crucial role in stabilizing endothelial junctions [19, 39–43]. However, distinct phenotypes of cell-cell junctions were identified in *heg1* and *krit1* mutants compared to *rasip1* mutants, suggesting diverse roles for the Rap1–KRIT1–HEG1–RASIP1 complex in regulating junctions. The cell-cell junctions in *rasip1* mutants appear fuzzy and diffuse across the presumed apical domains, while the junctions in *heg1* and *krit1* mutants are contorted and excessive [22, 31, 32]. Combined with our previous study showing that Rasip1 inhibits actomyosin contractility within the apical compartment and junctions, we identified distinct but complementary roles of Rasip1 and Krit1 in this complex, specifically in restricting and retaining contractility along junctions [31]. It has been reported that Rap1 activation leads to the recruitment of Rasip1 and Krit1 to junctions [19, 41]. The activation of Rap1 enables Rasip1 to localize to cell junctions, thereby inhibiting RhoA and its effector ROCK1 activity through ARHGAP29 [19, 20, 44]. Conversely, Rap1 enhances the localization of Krit1 to endothelial cell–cell junctions and promotes the interaction of ROCK2 with VE-cadherin–β-catenin as a scaffold protein [18, 41]. However, it remains largely unknown how junctional contractility is precisely controlled to maintain an appropriate level with these two seemingly counteractive partners. One possibility is that Rasip1 and Krit1 interact with distinct isoforms of ROCK in different cellular compartments [18]. Another possibility is that Rasip1 and Krit1 regulate contractility at different effective concentrations, with Krit1 predominating at lower concentrations to maintain junctional contractility, while Rasip1 predominates at higher concentrations, leading to contractility restraint under high tension. Further studies are required to explore these possibilities. Heg1 interacts with both Rasip1 and Krit1, potentially serving as a central regulatory hub. It has been demonstrated that Heg1 anchors Rasip1 to junctions but does not regulate the formation of the Rasip1-Rap1 or Rasip1-Radil-ARHGAP29 complexes [19]. This, combined with our previous study showing that the primary defects in *rasip1* mutants are due to increased apical contractility regulated by apical Rasip1, could potentially explain why *heg1* mutants display phenotypes similar to *krit1* mutants instead of *rasip1* mutants [31].

## Electronic supplementary material

Below is the link to the electronic supplementary material.

**Supplementary Material 1.**

**Supplementary Material 2: Video S1** (related to Figure 1B). Anastomosis and de novo lumen formation in zebrafish DLAV. Time-lapse series showing expression of ZO1-tdTomato (red) and GFP-Podxl1 (green), imaged from 30 hpf.

**Supplementary Material 3: Video S2** (related to Figure 1E). Oscillatory actomyosin cytoskeleton along the junctional ring. Time-lapse series showing expression of mRuby2-UCHD (red) and Myl9a-GFP (green), imaged from 32 hpf.

**Supplementary Material 4: Video S3** (related to Figure 1H). Actomyosin contraction straightens zigzag junctions. Time-lapse series showing expression of ZO1-tdTomato (red) and Myl9a-GFP (green), imaged from 32 hpf.

**Supplementary Material 5: Video S4** (related to Figure 3F). Mosaic labelling of tip cells in krit1 mutants displaying twisted junctions. Time-lapse series showing expression of Cdh5-Venus (green) and mosaically labelled GFP (red), imaged from 30 hpf.

**Supplementary Material 6: Video S5** (related to Figure 3G). 3D reconstruction of Figure 3G showing expression of Cdh5-Venus (green) and mosaically labelled GFP (red).

**Supplementary Material 7: Video S6** (related to Figure 4C). The expansion of luminal pockets between stalk and tip cells and the establishment of new luminal pockets between tip cells in WT embryos. Time-lapse series showing expression of Cdh5-Venus (green), imaged from 30 hpf.

**Supplementary Material 8: Video S7** (related to Figure 4D). The formation of zigzag and “∞”-shaped junctions in heg1 mutants. Time-lapse series showing expression of Cdh5-Venus (green), imaged from 30 hpf.

**Supplementary Material 9: Video S8** (related to Figure 4E). The formation of zigzag and “∞”-shaped junctions in krit1 mutants. Time-lapse series showing expression of Cdh5-Venus (green), imaged from 30 hpf.

**Supplementary Material 10: Video S9** (related to Figure 4G). Recruitment of Heg1-GFP to local regions of junctions upon contractions. Time-lapse series showing expression of Heg1-GFP (green) and mRuby2-UCHD (red), imaged from 32 hpf.

**Supplementary Material 11: Video S10** (related to Figure 5D). Twisted junctions untied in WT embryos with enriched Myosin. Time-lapse series showing expression of Cdh5-Venus (green) and Myl9a-GFP (red), imaged from 32 hpf.

**Supplementary Material 12: Video S11** (related to Figure 5G). Activation of opto-RhoA untied the twisted junctions in krit1 mutant. Time-lapse series showing expression of Cdh5-Venus (green) and RhoA-BcLOV4-mCherry (red), imaged from 32 hpf. Blue labels the ROI of activation.

**Supplementary Material 13: Video S12** (related to Figure 6C). Collapse of ectopically contorted luminal pockets in heg1 mutants. Time-lapse series showing expression of Cdh5-Venus (green), imaged from 33 hpf.

**Supplementary Material 14: Video S13** (related to Figure 6D). Collapse of ectopically contorted luminal pockets in krit1 mutants. Time-lapse series showing expression of Cdh5-Venus (green) and Rasip1-Scarlet-I (red), imaged from 33 hpf.

**Supplementary Material 15: Video S14** (related to Figure 6G). 3D reconstruction of lumenization at DLAV through “∞”-shaped junctions in WT embryos.

**Supplementary Material 16: Video S15** (related to Figure 6H). 3D reconstruction of transient lumenization at DLAV in a very small portion of krit1 mutants.

## Data Availability

Data is provided within the manuscript and supplementary information files.
