# Supplementary material for: Oscillatory contractile forces refine endothelial cell-cell interactions for continuous lumen formation governed by Heg1/Ccm1
Source: Angiogenesis. 2024 Sep 9;27(4):845–60. doi: 10.1007/s10456-024-09945-5 (PMC11564304; doi:10.1007/s10456-024-09945-5)
Supplement: Supplementary file 1 — Supplementary Material 1. [file 10456_2024_9945_MOESM1_ESM.docx]

**Supplemental information**

**Oscillatory Contractile Forces Refine Endothelial Cell-Cell Interactions for Continuous Lumen Formation Governed by Heg1/Ccm1**

**Jianmin Yin^1,*^, Ludovico Maggi^1^, Cora Wiesner^1^, and Markus Affolter^1,*^, Heinz-Georg Belting^1,2,*^**

**Supplementary Note**

**Figure S1**, related to Figure 4

**Figure S2**, related to Figure 5

**Figure S3**, related to Discussion

**Video S1,** related to Figure 1B

**Video S2,** related to Figure 1E

**Video S3,** related to Figure 1H

**Video S4,** related to Figure 3F

**Video S5,** related to Figure 3G

**Video S6,** related to Figure 4C

**Video S7,** related to Figure 4D

**Video S8,** related to Figure 4E

**Video S9,** related to Figure 4G

**Video S10,** related to Figure 5D

**Video S11,** related to Figure 5G

**Video S12,** related to Figure 6C

**Video S13,** related to Figure 6D

**Video S14,** related to Figure 6G

**Video S15,** related to Figure 6H

**Supplementary Note 1: Molecular biology and transgenesis**

**1.1** **Cloning and transgenesis of *Tg(UAS:Heg1-GFP)^ubs61^***

The cDNA of *heg1* was cloned from zebrafish cDNA library into pME vector through restriction cloning with the MfeI and XbaI sites.

heg1-MfeI-F: TCGTTTCTCAATTGatgatggaaacgtgcgctc

heg1-XbaI-R: ACATATCGTCTAGATtcaaaagtagtctcttcggcgtg

The BamHI and NotI restrictive sites were incorporated via PCR at the 5’ end of the EGF-like domain within the middle of extracellular region of Heg1.

heg1-MI-notI-F: TGCTCTGAGCGGCCGCccaggtcatgtatgtggacc

heg1-MI-BamHI-R: GCTAAGAGGGATCCtgatgctgatgttctaagaggcatc

EGFP was integrated at the 5' end of the EGF-like domain of Heg1, utilizing the pre-existing BamHI and NotI restriction sites.

GFP-BamHI-F: GAGTGTAGGGATCCGGCATGGTGAGCAAGGGCGAG

GFP-NotI-R: TTATCCAAGCGGCCGCCGCTTCCTCCTCCGCTTCCTCCcttgtacagctcgtccatgc

The constructed *pME-Heg1-GFP* was fully sequenced. The *pME-Heg1-GFP* was cloned into PDestTol2CG2 through the LR reaction of gateway cloning with a 4x UAS promoter and 3' poly A tail.

**1.2** **Cloning and transgenesis of *Tg(UAS:*** ***GFP-Radil2a)^ubs62^***

The cDNA of *Radil2a* was cloned from zebrafish cDNA library into pME vector through restriction cloning with the MfeI and XbaI sites.

radilb-MfeI-F: TCAACCTTCAATTGCTCTGAGCGGCCGCatgatttcggaggaaaggagc

radilb-XbaI-R: CACACCGTTCTAGAttagcatctggtgatgcagc

The primer radilb-MfeI-F includes the NotI restriction site for subsequent EGFP insertion at the N-terminus of Radil2a. EGFP was integrated at the 5' end of the Radil2a, utilizing the pre-existing MfeI and NotI restriction sites.

GFP-MfeI-F: ATACGTGGCAATTGATGGTGAGCAAGGGCGAG

GFP-NotI-R: TTATCCAAGCGGCCGCCGCTTCCTCCTCCGCTTCCTCCcttgtacagctcgtccatgc

The constructed *pME-GFP-Radil2a* was fully sequenced. The *pME-GFP-Radil2a* was cloned into PDestTol2CG2 through the LR reaction of gateway cloning with a 4x UAS promoter and 3' poly A tail.


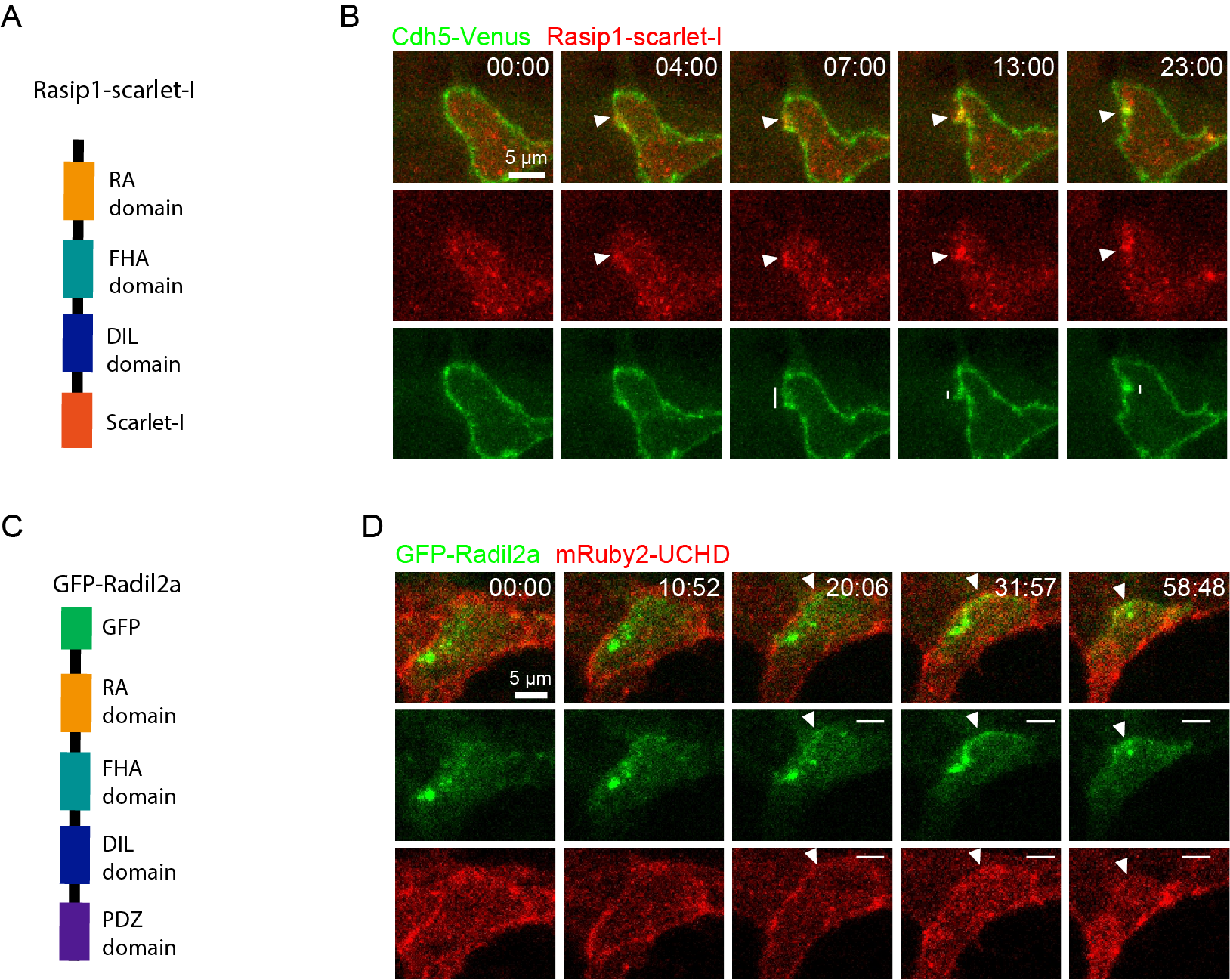


Fig. S1 Local enrichment of Rasip1 and Radil2a at constricting junctions. (A) Schematic representation of recombined Rasip1-scarlet-I. (B) Time-lapse imaging of Cdh5-Venus and Rasip1-scarlet-I, showing local enrichment of Rasip1 at constricting junctions. White arrowheads mark locally enriched Rasip1-scarlet-I. (C) Schematic representation of recombined GFP-Radil2a. (D) Time-lapse imaging of Cdh5-Venus and GFP-RadilB, indicating local enrichment of RadilB at constricting junctions. White arrowheads highlight locally enriched GFP-RadilB.


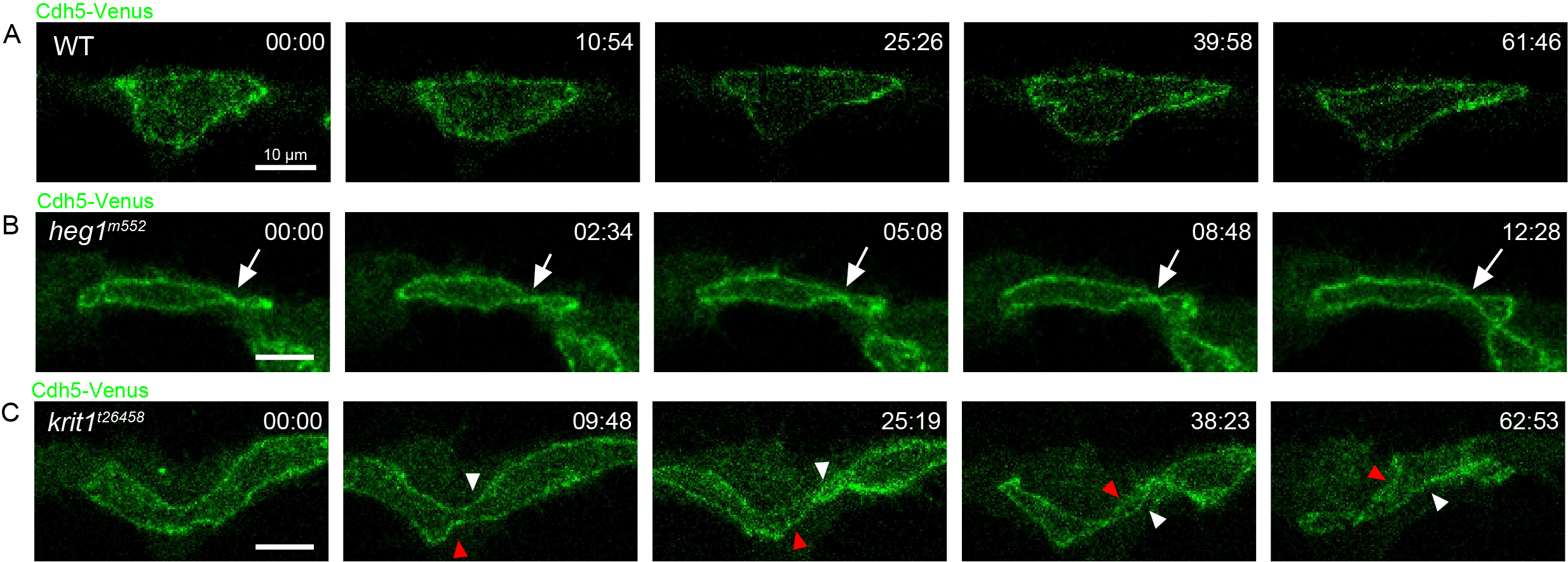


Fig. S2 Ectopic twisting of junctional rings in *heg1* and *krit1* mutants. (A-C) Time-lapse imaging of Cdh5-Venus in wild-type embryos (A), *heg1* mutants (B) and *krit1* mutants (C), demonstrating ectopic twisting of circular junctional rings in *heg1* mutants (B) and *krit1* mutants (C). White arrows denote the crossing point between two loops. White and red arrowheads indicate opposite sides of a junctional ring moving towards each other (C).


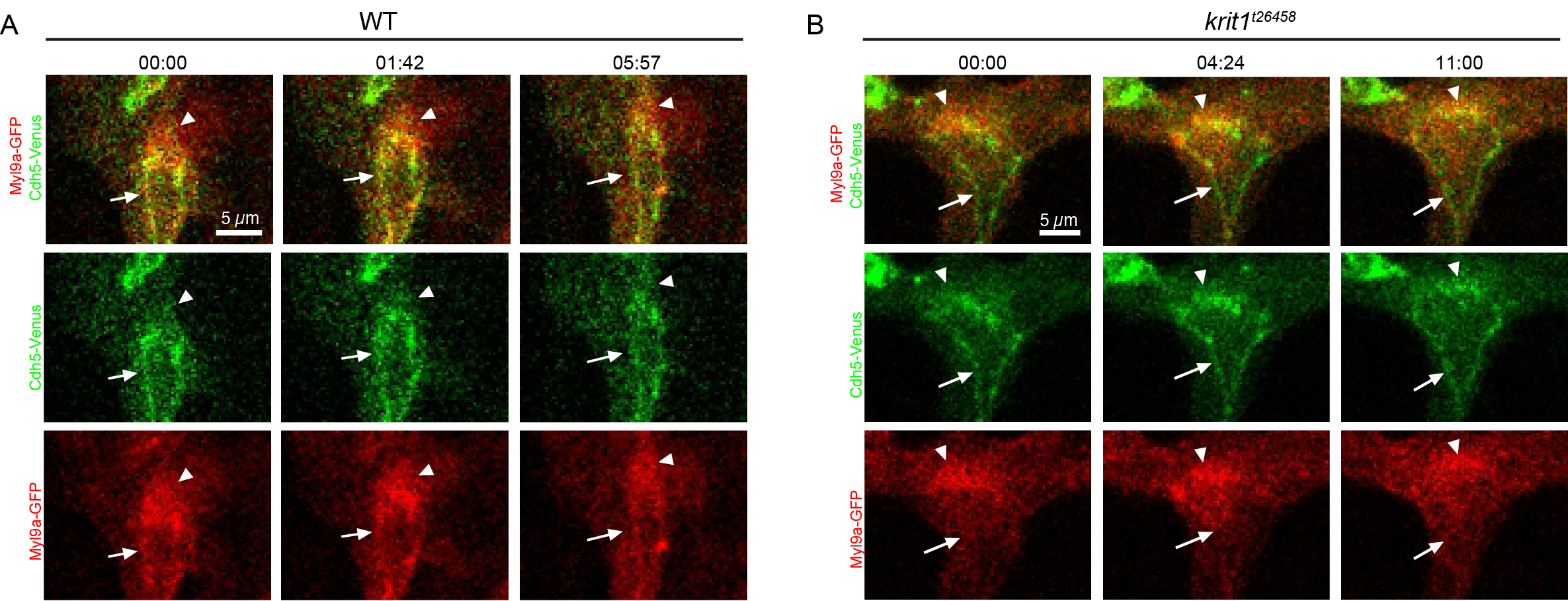


Fig. S3 The distal population of myosin is independent of Ccm1. (A and B) Time-lapse imaging of expanding junctional rings labelled with Cdh5-Venus and Myl9a-GFP in wild-type embryos (A) and *krit1^t26458^* mutants (B). White arrowheads indicate the distal Myl9a outside of the junctional rings. White arrows indicate the junctional Myl9a that was present in wild-type embryos but largely absent in *krit1^t26458^* mutants.

**Supplementary Video legends**

**Video S1 (**related to Figure 1B). Anastomosis and *de novo* lumen formation in zebrafish DLAV. Time-lapse series showing expression of ZO1-tdTomato (red) and GFP-Podxl1 (green), imaged from 30 hpf.

**Video S2 (**related to Figure 1E). Oscillatory actomyosin cytoskeleton along the junctional ring. Time-lapse series showing expression of mRuby2-UCHD (red) and Myl9a-GFP (green), imaged from 32 hpf.

**Video S3 (**related to Figure 1H). Actomyosin contraction straightens zigzag junctions. Time-lapse series showing expression of ZO1-tdTomato (red) and Myl9a-GFP (green), imaged from 32 hpf.

**Video S4 (**related to Figure 3F). Mosaic labelling of tip cells in *krit1* mutants displaying twisted junctions. Time-lapse series showing expression of Cdh5-Venus (green) and mosaically labelled GFP (red), imaged from 30 hpf.

**Video S5 (**related to Figure 3G). 3D reconstruction of Figure 3G showing expression of Cdh5-Venus (green) and mosaically labelled GFP (red).

**Video S6 (**related to Figure 4C). The expansion of luminal pockets between stalk and tip cells and the establishment of new luminal pockets between tip cells in WT embryos. Time-lapse series showing expression of Cdh5-Venus (green), imaged from 30 hpf.

**Video S7 (**related to Figure 4D). The formation of zigzag and “∞”-shaped junctions in *heg1* mutants. Time-lapse series showing expression of Cdh5-Venus (green), imaged from 30 hpf.

**Video S8 (**related to Figure 4E). The formation of zigzag and “∞”-shaped junctions in *krit1* mutants. Time-lapse series showing expression of Cdh5-Venus (green), imaged from 30 hpf.

**Video S9 (**related to Figure 4G). Recruitment of Heg1-GFP to local regions of junctions upon contractions. Time-lapse series showing expression of Heg1-GFP (green) and mRuby2-UCHD (red), imaged from 32 hpf.

**Video S10 (**related to Figure 5D). Twisted junctions untied in WT embryos with enriched Myosin. Time-lapse series showing expression of Cdh5-Venus (green) and Myl9a-GFP (red), imaged from 32 hpf.

**Video S11 (**related to Figure 5G). Activation of opto-RhoA untied the twisted junctions in *krit1* mutant. Time-lapse series showing expression of Cdh5-Venus (green) and RhoA-BcLOV4-mCherry (red), imaged from 32 hpf. Blue labels the ROI of activation.

**Video S12 (**related to Figure 6C). Collapse of ectopically contorted luminal pockets in *heg1* mutants. Time-lapse series showing expression of Cdh5-Venus (green), imaged from 33 hpf.

**Video S13 (**related to Figure 6D). Collapse of ectopically contorted luminal pockets in *krit1* mutants. Time-lapse series showing expression of Cdh5-Venus (green) and Rasip1-Scarlet-I (red), imaged from 33 hpf.

**Video S14 (**related to Figure 6G). 3D reconstruction of lumenization at DLAV through “∞”-shaped junctions in WT embryos.

**Video S15 (**related to Figure 6H). 3D reconstruction of transient lumenization at DLAV in a very small portion of *krit1* mutants.
